# Pesticide Knowledge and Safety Practices among Farm Workers in Kuwait: Results of a Survey

**DOI:** 10.3390/ijerph14040340

**Published:** 2017-03-24

**Authors:** Mustapha F.A. Jallow, Dawood G. Awadh, Mohammed S. Albaho, Vimala Y. Devi, Binson M. Thomas

**Affiliations:** Environment and Life Sciences Research Center, Kuwait Institute for Scientific Research, P.O. Box 24885, Safat 13109, Kuwait; dawadh@kisr.edu.kw (D.G.A.); mbahouh@kisr.edu.kw (M.S.A.); ydevi@kisr.edu.kw (V.Y.D.); bmthomas@kisr.edu.kw (B.M.T.)

**Keywords:** pesticide exposure, knowledge, risk, health hazards, smallholder farmers

## Abstract

The unsafe and indiscriminate use of pesticides in agriculture represents a major hazard to the environment and human health. The aim of this study was to assess the levels of knowledge, attitude and practices of Kuwaiti farmers regarding the safe use of pesticides. A total of 250 farmers participated in this study through in-depth interviews and observations on-farm. The majority of the farmers acknowledged that pesticides were harmful to their health (71%) and the environment (65%). However, farmers’ level of knowledge of pesticide safety is insufficient. Over 70% of the farmers did not read or follow pesticide label instructions, and 58% did not use any personal protective equipment (PPE) when handling pesticides. Educated farmers were significantly more likely to use PPE compared with famers with limited formal education (χ^2^ = 9.89, *p* < 0.05). Storage of pesticides within living areas was reported by 20% of farmers. When disposing of pesticide wastes, respondents adopted unsafe practices such as discarding, incinerating, or burying empty pesticide containers on-farm, or reusing the containers. Farmers also reported disposing leftover pesticide solution or old pesticide stocks on-farm or in the sewer. A significant number (82%) of the farmers reported at least one symptom of acute pesticide poisoning. Although farmers’ knowledge of pesticide hazards was high, the reported safety measures were poor. Comprehensive intervention measures to reduce the health and environmental risks of pesticides are needed, including pesticide safety training programs for farmers, stringent enforcement of pesticide laws, and promoting integrated pest management and non-synthetic methods of pest control.

## 1. Introduction

Pesticides have become an integral part of present day farming, and play a major role in increasing agricultural productivity. However, the indiscriminate and extensive use of pesticides represents one of the major environmental and public health problems all over the world [1,2]. If improperly used, pesticides can lead to secondary pest outbreaks [3], destruction of non-target species [4], soil, water, and air contamination [5,6], and residues in primary and derived agricultural products [7] that endanger both the environment and human health. Farm workers’ exposure to pesticides has been associated with adverse health effects like cancer and birth defects resulting in hundreds of fatalities, the majority of which occur in developing countries [8,9]. Farmers, and especially those directly involved in the handling of pesticides, are at a high risk of exposure to pesticides through contact with pesticide residues on treated crops, unsafe handling, storage and disposal practices, poor maintenance of spraying equipment, and the lack of protective equipment or failure to use it properly [8,10].

These risks may be exacerbated by lack of information on pesticide hazards [10], the perception and attitude of farmers regarding risk from pesticide exposure [11], and to lack of education and poor knowledge and understanding of safe practices in pesticide use, including storage, handling and disposal [12]. Higher levels of education gives pesticide users better access to information and more knowledge of the risks associated with pesticides, and how to avoid exposure. While less educated farmers may be hampered in their ability to understand the hazard warnings on pesticide labels, how to avoid exposure, and how to follow recommended safety and application guidelines. For example, illiteracy and lack of knowledge on the extent to which pesticides represent a hazard have been considered the most important barriers for the adoption of self-protective behaviors by farmers, in particular the use of personal protective equipment (PPE) [13,14].

Kuwait has made significant investments in the past four decades towards developing new agricultural strategies, and has created a favorable environment for agricultural expansion to help facilitate at least a modest level of self-sufficiency in food production. Consequently, there is a growing interest in agricultural activities, especially vegetable production in open fields and protected environments for fresh market consumption. The prevalence of the problems caused by insect pests and diseases has resulted in high demand for pesticides in Kuwait [15]. For most farmers, vegetable production, especially in greenhouse environments, is not possible without intensive use of chemical pesticides due to farmers’ lack of access to non-synthetic methods of pest control. The annual consumption of pesticides in Kuwait was about 4.5 (kg ai)/ha per year in 2007 [16], and by 2015 this figure has increased to 12.8 (kg ai)/ha per year [17]. This excessive amount of pesticide use does not translate into increased crop yields for the farmers, but rather increases the potential to adversely affect human health and the environment. Pesticide use is further complicated by farmers’ not perceiving these chemicals as hazardous or that they have to be handled correctly.

Due to the potential health effects of pesticides, most countries, including Kuwait, have developed laws and regulations to encourage safe pesticide use. For example, Law No. 21 of 2009 and Law No. 42 of 2014, dealing with procedures for the use of pesticides including, registration, sale, distribution, transportation, storage and handling. Similarly, resolution No. 95 of 1995 is concerned with banning the circulation of hazardous pesticides to protect human health and the environment. However, the basic problem lies in the inadequacy of enforcing these laws at the retail and farm level due to the lack of effective enforcement mechanisms. Evidence exists of pesticide-related health effects in Kuwait [18,19,20]. Pesticide residues have been detected in a number of vegetables in Kuwait [21,22,23,24], and the presence of chlorinated pesticides in the breast milk of lactating women has raised even greater concerns about possible health risks to breastfed infants [25].

Research on pesticides in Kuwait has primarily focused on pesticide toxicity, the effects of pesticides on human health and the environment [22,24], and more recently the determinants of pesticide overuse by farmers [17]. Jallow et al. [17] reported that 58% of smallholder vegetable farmers in Kuwait overused pesticides. Pesticide application frequency ranged from two times a month up to once a week, depending on the crop. In this paper, we focus specifically on the understanding of farmers’ knowledge and practices with respect to pesticide safety. Understanding farmers’ knowledge of pesticides and safety practices is vital not only for identifying exposure situations and knowledge gaps, but also to provide valuable information that can contribute to educational and policy recommendations aimed at preventing or reducing the health and environmental hazards associated with pesticides. Therefore, the aim of the work described in this paper is to assess the levels of knowledge, attitude and practices of Kuwaiti smallholder farmers regarding the safe use of pesticides. Specifically we aim to:

1. Determine the knowledge and awareness of the farmers regarding pesticides, and their experiences with acute pesticide poisoning in connection with handling pesticides.
2. Assess farmers’ practices and attitudes regarding storage, handling and disposal of pesticides.
3. Evaluate the protective measures taken by farmers, including the adoption of PPE, to reduce occupational pesticide exposure.

## 2. Materials and Methods

### 2.1. Questionnaire Development and Delivery

Diagnostic surveys, formal interviews, and field observations were used to gather information on farmers’ knowledge of pesticides and safety practices. Data were collected through face-to-face interviews with 250 farmers (farm managers and their employees directly involved in pesticide use and management), from 200 farms in Kuwait’s two major agricultural regions (Wafra and Abdally). Detailed description of the study area, including cropping systems is highlighted in Jallow et al. [17]. The sample size (200 farms) was determined using the Leslie Kish [26] formula to provide a good estimate of the sampled population. Wafra and Abdally regions account for more than 90% of the total crop production in Kuwait [27]. Cropping systems are mainly based on open field and greenhouse production, with about 30,000 ha under cultivation. Farms of about 5 ha dominate the agricultural landscape, mostly growing dates, vegetables, cereals, pulses, and forages. It is worth noting that the majority of the farms, although owned by Kuwaitis, are typically operated and managed by predominantly expatriate laborers from North Africa and the Indian subcontinent who also constitute the majority of the agricultural labor force in Kuwait [27]. Multistage-sampling was used for the purpose of the survey. Each region was divided into four clusters, and within each cluster individual farms were selected by random sampling using an automatic random number generator. In all, 120 farms from Abdally and 80 farms from Wafra were selected for the survey.

A questionnaire was designed in English and translated into Arabic, the national language that is understood by majority of the farmers, and was administered either in Arabic or English on-farm. The questionnaire included closed and open-ended questions, and was pre-tested by randomly interviewing twenty farmers not included in this study. The closed questions were in a multiple choice format so that respondents had to select only the appropriate answer or answers that they thought best described their opinion or attitude on a particular issue. The questionnaire contained three main sections. The first section was designed to collect information on personal characteristics of the farmers including age, educational level, and years of farming experience. The second section focused on collecting information on farmers’ level of awareness of pesticide laws and regulations, and knowledge and understanding of pesticides with respect to the environmental and human health. In addition, we also collected data on self-reported toxicity symptoms associated with pesticide use, as well as farmers’ knowledge about exposure routes. When symptoms were assessed, respondents were asked if within the past year prior to the date of the interview they experienced at least one heath impairment immediately after applying or handling pesticides. If the answer was yes, respondents were asked to specify which symptoms they had experienced. The third section included questions regarding pesticide handling and safety practices including reading and following label instructions, storing and disposing of pesticides and empty containers, and use of PPE and other protective practices during and after pesticide application.

### 2.2. Data Analysis

All data were coded, entered, and then analyzed using SPSS version 20 (SPSS Inc., Chicago, IL, USA) and Microsoft Office Excel 2010 (Microsoft Corporation, Redmond, WA, USA). Descriptive results were expressed as frequencies and percentages, and the Chi-square test (χ^2^) was used to measure the possible association between nominal variables [28]. We used α ≤ 0.05 as a criterion for statistical significance.

## 3. Results

### 3.1. Demographic Characteristics and Profile of the Farmers

All farmers surveyed in the study were men, as farming activities especially those related to pesticide use are performed exclusively by men in Kuwait. The majority of the farmers (69.70%) interviewed were between 21 and 40 years old, with the average age being 29.8 years. Slightly more than 9% of the farmers were 50 years and over. A considerable number of the farmers (16.8%) were illiterate or had not completed elementary education, 69.2% had completed either elementary or secondary education, and 14% were educated to a tertiary level. The majority of the farmers educated to the tertiary level were expert agronomists (71.4%), and 22.9% had received training in pest management. Most of the respondents (68%) had 5–10 years of farming experience, with 10.4% having more than 10 years of farming experience. Farm sizes varied from less than 5 ha to over 10 ha, with an average of 4.5 ha.

### 3.2. Pesticides Used

A total of 76 pesticide active ingredients were found to be in use during the survey period (Table 1). About 47% (35 out of 76) of the pesticides used belonged to the World Health Organization (WHO) toxicity class II (moderately hazardous), with a few (9%) under toxicity class Ib (highly hazardous). WHO toxicity class II pesticides (moderately hazardous) were used by 90% of the farmers, followed by class III (slightly hazardous) 68%. Slightly more than 12% of the farmers used class Ib pesticides (highly hazardous), including monocrotophos, oxamyl, abamectin, beta-cyflurthrin, methomyl, fenamiphos, triazophos, and fenamiphos. Insecticides were used by 98% of the farmers, followed by fungicides and bactericides (79%), nematicides (24%) and herbicides (5%).

### 3.3. Farmers’ Knowledge, Attitude, and Understanding about Pesticides

The farmers’ level of knowledge of pesticides, including exposure routes, effects on the environment and human health, and their awareness of pesticide laws and regulations was analyzed in Table 2. The majority (64%) of the farmers had not received any training or technical support on the judicious use and safe handling of pesticides, while 36% were trained. Although most farmers agreed that pesticide use poses some risk to human health (71%) and the environment (65%), they also indicated that pesticides were indispensable for high crop yield (80%). Over 70% of the farmers did not read or follow instructions on pesticide labels, because they were unable to read and understand the meaning of the labels (56%), the labels were written in English (a foreign language to them) (35%), and the instructions were too long and complicated (45%). Slightly more than 15% of the farmers indicated that font sizes on the labels were too small to easily read. Only 28% of the farmers were able to read, understand, and follow pesticide label instructions correctly.

When farmers were asked to indicate how pesticides enter the human body, dermal (54%), inhalation (86%), and oral (42%) were stated as the most common routes of exposure to pesticides. About 14% of the farmers indicated lack of knowledge of any route of exposure to pesticides. The majority of the farmers (58%) are aware that some pesticides have been banned or are restricted for use. However, among this group, only 20% of the farmers knew the names of some the pesticides that are banned or restricted for use. Examples include dichloro-diphenyl-trichloroethane (DDT), permethrin, methiocarb, and methamidophos, all of which are highly hazardous to human health and the environment. Among farmers who are aware that some pesticides have been banned or restricted for use, the high toxicity of the pesticides (58%), not effective (79%) and expensive (28%) were cited as major reasons for the ban or restriction.

### 3.4. Farmers’ Practices on Storage and Disposal of Pesticides

Farmers’ attitudes towards storing of pesticides and disposal of residual pesticide solutions, expired pesticides, and empty pesticide containers are shown in Table 3. The majority of the farmers (59%) stored their pesticides in locked chemical stores designated only for pesticides. Respondents also stored their pesticides in open sheds just for pesticides (34%) and in the open field (30%). A worrying 15% of the farmers reported storing pesticides in an animal house, in a refrigerator with other items (8%), or within their living area (20%). Respondents with higher education levels were significantly less likely to store pesticides in their home (χ^2^ = 20.89, *p* < 0.01). When asked what they do with residual pesticide solutions, 82% of respondents reported that they apply the leftover solution on other crops, disposed the solution in the field (35%), in the sewer (4%) or deliver the solution to the municipality hazardous waste collection sites for disposal (23%). Only 18% of respondents reported mixing only the amount of pesticides that is needed for the application at hand.

Disposal of old pesticide stocks is often done by delivering them to the municipality hazardous waste collection sites (16%), disposing in open fields (27%), in the sewer (3%) or returning the obsolete pesticide to pesticide retailers (5%). Over 60% of the farmers claimed that they buy only the amount of pesticides they need. The common way of disposing of empty pesticide containers was placing them in garbage containers and/or dumpsters for disposal at the landfill (50%), incinerating them on the farm (43%), or delivering them to the municipality hazardous waste collection sites for disposal (39%). Respondents (25%) also buried the containers on-farm or discarded them on the farm (27%). Alarmingly, 6% of the farmers reported re-using empty pesticide containers for household purposes. A significant association (χ^2^ = 8.98, *p* < 0.05) was observed between years of farming experience and safe pesticide storage and disposal.

### 3.5. Farmers’ Safety Practices against Occupational Exposure to Pesticides

Protective measures during and after pesticide application are important to reduce exposure to pesticides. The majority (58%) of the farmers did not use any PPE when mixing or spraying pesticides (Table 4). When respondents were asked to indicate the main reasons for not using PPE, lack of availability when needed (35%), and PPE being uncomfortable in the local hot and humid climate (90%), too expensive (65%), and slowing you down (29%) were the most reasons cited. Respondents (6%) also cited not experiencing any health problems from using pesticides as reason for not using PPE. Among respondents who reported using PPE, less that 5% wore all the recommended six key PPE items (coveralls, protective boots, glasses/goggles, gloves, respirator, and hat). The PPE most often used were protective gloves (61%), hats (42%), and glasses/goggles (48%). A significant number of respondents reported not wearing respirators (70%), coveralls (68%), or protective boots (54%) at all. Younger and educated farmers were more likely to use PPE compared with older farmers or famers with limited formal education (χ^2^ = 9.89, *p* < 0.05). However, no association was observed between farming experience and use of PPE.

Apart from PPE use, farmers were asked if they implement other safety measures to reduce their risk of exposure to pesticides. The majority of respondents reported not eating (72%), drinking (65%) or smoking (59%) when mixing or applying pesticides (Table 5). Furthermore, 82% of respondents reported taking a shower immediately after mixing or spraying pesticides. Over 70% of respondents, however, did not wash work clothing used while mixing or spraying pesticides separately from other cloths. Similarly, 46% of respondents reported that they did not consider wind direction when spraying pesticides.

### 3.6. Self-Reported Toxicity Symptoms Related to Pesticides

A significant number (82%) of the farmers reported at least one symptom of acute pesticide poisoning in the previous year immediately after applying or handling pesticides, while 18% of respondents did not ascribe any health problem encountered to pesticide exposure (Table 6). The most frequently reported symptoms were headaches (82%), skin irritation (58%), nausea (49%), itchy eyes (79%), dizziness (41%), fatigue (50%), and coughing (22%). Other symptoms reported by respondents were poor vision, stomach ache, excessive sweating, shortness of breath, and vomiting. When respondents were asked what action they took following an incident of poisoning, a majority (75%) reported taking no action as the incident was minor or required only self-medication. Only 5% of respondents reported a serious poisoning incident that required medical attention in a hospital.

## 4. Discussion

Understanding farmers’ level of knowledge and practices regarding the safe use of pesticides is vital for providing sound educational and policy strategies that aim at limiting the health and environmental hazards caused by pesticides. The majority of farmers in this study were well aware of the harmful effects of pesticides with regard to the environment and human health, but contrary to expectations, this did not significantly change their practices or attitudes towards safe pesticide use. This suggests that even though farmers may know the hazards of pesticides very well, they may often adopt risky behaviors because of lack of education and poor knowledge and understanding of safe practices in pesticide use [10], or they are more concerned with high economic returns from their crops than with their own health [29]. A considerable number of the respondents in this study were illiterate or had limited formal education, and did not receive any training or technical support in pesticide safety. Consequently, these farmers are hampered in their ability to read and understand pesticide labels regarding the correct and safe use of pesticides, or written communication about how to avoid the risks of exposure [30]. The fact that the majority of the farmers (72%) indicated that they did not read or follow pesticide labels (Table 2) is a great cause for concern, and perhaps indicates a general ignorance of the importance of pesticide labels in reducing exposure risk. Educated farmers are more knowledgeable about pesticide safety, have better ability to read, understand and follow hazard warnings on labels, and conceptualized the consequences of poor pesticide usage practices [12]. Even when able to read, some respondents in this study acknowledged they were reluctant to read pesticide labels because of their experience with pesticide use.

Our study showed some worrying practices about storage of pesticides, with 20% of the farmers storing pesticides in living areas or in refrigerators (8%) with other items (Table 3). This demonstrates the farmers’ lack of knowledge of pesticides and the appropriate approach for storing pesticides. Storing pesticides in living areas can increase the potential for high exposure, especially when these areas are the places where farmers prepare food, eat, and sleep. Farmers also stored pesticides in animal housing that could pose a danger to farm animals (Table 3). Unfortunately, storing of pesticides in residences and in the open is prevalent in many developing countries. Matthews [10] reported that 27% of 8500 smallholder farmers in 26 countries stored pesticides in the home or in open areas, and nearly half indicated that they rarely or never locked pesticides away. These risky behaviors can be attributed to farmers’ lack of technical knowledge and training on safe pesticide use. Educated farmers and farmers who have access to training on pesticide use are more likely to store pesticides in locked stores designated for pesticides [31]. Likewise, they are more likely to be aware of pesticide-related adverse health and environmental effects [32].

Farmers in this study generally demonstrated a poor knowledge of pesticide disposal, and more than 80% of respondents re-spray treated areas with leftover pesticide solutions. Leftover pesticide solutions or old pesticide stock were also disposed of in the field or in the sewer (Table 3). These poor pesticide handling practices can lead to harmful residues in harvested produce, and soil and water contamination, posing a threat to both human and environmental health [11]. Some respondents reported that they only mixed the amount of pesticides needed or they deliver leftover solutions to hazardous waste collection sites for disposal, both good options for reducing the hazards of pesticides (Table 3). Unfortunately, these farmers represented less than 25% of all farmers interviewed in this study. Unsafe disposal of pesticide containers may be an important source of pesticide exposure [31]. When disposing of empty pesticide containers, the majority of respondents adopted unsafe behaviors such as discarding, incinerating or burying containers on-farm. These practices may lead to environmental contamination and a risk to human health, and have been reported as a major problem in a number of studies [10,33]. About 5% of the farmers indicated that they reuse the empty pesticide containers for household purposes, increasing their chances of exposure to pesticides. Respondents emphasize that they always wash the empty pesticide containers before reuse, and they reinforce this by saying that there is no exposure risk because they do not smell the pesticides. Evidence of farmers’ reuse of empty pesticide containers has been reported in other studies [34].

Use of appropriate PPE, such as coveralls, and the adoption of other protective measures and good personal hygiene such as showering, not smoking, eating or drinking while handling pesticides are considered good practices to reduce occupational pesticide exposure [10]. An increase in the use of protective measures decreases the probability of poisoning by 44% to 80% [35], whereas lack of PPE use increases the potential for dermal and respiratory exposure to pesticides [36]. An important finding in this study is that there are low levels of adoption of protective behaviors to reduce occupational exposure to pesticides. The majority of farmers fail to use any PPE, while fewer than 5% use all the recommended equipment (Table 4). The main reason mentioned for not using PPE was the discomfort under hot and humid conditions, as the environment in Kuwait is characterized by high ambient temperatures, with summer temperatures exceeding 45 °C [37]. This indicates that farmers may be more willing to risk exposure to pesticides than to use PPE under such climatic conditions [38]. Lack of PPE use is made worse by some farmers not spraying according to the wind direction, and drink, eat and smoke when handling pesticides, all of which increase the danger of poisoning [10]. Similarly, they sprayed more often than required, up to once a week depending on the crop, using pesticides highly hazardous to human health and the environment [27]. Education status and training in pesticide use and safety are strong determinants of the appropriate use of PPE. For example, the perception that PPE were useful to prevent exposure to pesticides was associated with at least a high school education among migrant farm workers in USA [39], farmers in India [40] and in Mexico [14].

Findings in this study will enable government and regulatory agencies to make better-informed decisions and policy recommendations aimed at preventing or reducing the health and environmental hazards associated with pesticides. The knowledge gaps identified in this study could also be used to designed knowledge-based training programs for farmers. Participation in training programs leads to increased levels of knowledge about safety precautions while handling pesticides [29,41]. It is imperative that proper training programs on pesticide safety and on the hazards of pesticide exposure be developed to address gaps in farmers’ knowledge about pesticides. The national agricultural extension service should play a pivotal role in the training of farmers, and the information they provide should be up-to-date, accurate, and easy to understand to inspire confidence and trust among the farmers. Farmers’ source of information and the degree to which they trust the informants may shape their perceptions of risk of pesticides and the adoption of preventative measures [42]. Our study pointed to significant illiteracy rates among farmers, and this represents a challenge in the design of appropriate training programs. Given that most farmers are unable to read and write, a more interactive and participatory training model is required, for example, by using pictograms to simplify pesticide labels and communicate risk information. The pictograms should be unambiguous and easy to understand, to prevent misinterpretations of the risk information. Using pictograms is seen as a key element for overcoming literacy challenges in transmitting pesticide risk information [43]. Knowledge of the farmers can further be reinforced through field demonstrations. Such capacity-enhancement activities will provide farmers the opportunity to learn and understand how to handle pesticide safely, and how to adopt preventative measures to reduce the risk against occupational exposure. It is also important to educate farmers about alternative cropping systems that are less dependent on pesticides, while at the same time promoting integrated pest management practices.

Pesticide retailers play a critical role in the dissemination of agricultural information to farmers, and farmers in Kuwait rely on them as the primary information and knowledge source on pesticides [17]. Retailers can downplay environment and health effects of pesticides when dealing with farmers for greater profit-making [44], or may be constrained by the lack of knowledge of pesticides [45]. Therefore, training pesticide retailers to increase their knowledge of pesticide safety and risk communication is critical. Retailers should have, at minimum, a technical advisor with competence in the handling of pesticides and knowledge of their hazards to appropriately advise farmers and other end-users. Lack of training of pesticide retailers, who may serve as advisors to farmers and other end-users, has been considered a key factor contributing to occupation exposure to pesticides [46]. Promoting safe pesticide use will also require educating farmers and pesticide retailers about the pesticide regulatory framework in Kuwait, and the obligations currently in place. Some farmers lack any knowledge of the pesticide regulatory framework, and were completely unaware that some pesticides are banned or restricted for use because of their toxicity (Table 2). This lack of knowledge is likely to contribute to increased risk to the farmers.

Training farmers alone may not be sufficient to reduce the risk posed by pesticides. Interventions that provide farmers with information and knowledge of pesticide safety should be complemented with other strategies. Greater priority must be given to enforcing existing pesticide laws and regulations at the retail and at the farm level, through surveillance and monitoring activities. Ideally, there should be a pesticide safety certification program for pesticide applicators and retailers to ensure that only those certified are allowed to sell, handle, or apply pesticides. It is also critical to raise awareness among the general public, who may be directly or indirectly be exposed to pesticides, about the risk of these chemicals. Risks to the general public may include inadvertent dermal and inhalation exposure from sprayed fields, contact with treated crops during farm visits, or through harmful residues in primary and derived agricultural products [47].

A limitation of a study such as this is that it is based mainly on self-reported data, relying on the honesty of respondents which is subjected to bias [48]. As a self-report, it is possible that there may be some inaccurate data such as respondents wanting to report socially desirable behaviors. For example, self-report of PPE use and safe disposal of empty pesticide containers, and the adoption of other safety practices may be influenced by the respondents’ desire to indicate that they comply with protective measures against occupational pesticide exposure. However in some farms, observations were conducted to verify farmer reports, and we often observed farmers discarding empty pesticide containers or incinerating them on-farm. These observations were supported by the responses of the interviewed farmers. Another limitation relates to the inability to directly link health symptoms experienced by respondents to pesticide exposure. The health symptoms experienced by respondents, such as headaches and fatigue, were not specific, and in some of the cases these symptoms might have been due to causes other than exposure to pesticides, such as long exposure to the sun, especially if no head protection is worn. Nevertheless, that the symptoms reported by respondents occurred immediately after applying or handling pesticides and the frequency of occurrence is a great cause of concern. Finally, based on the number of respondents (250 farmers), we cannot claim that the results are representative for all Kuwaiti farmers. It was not feasible to interview all farmers in Kuwait. However, our intention is not to generalize, but to explore and highlight important occupational health and pesticide safety issues for individual farmers. Furthermore, our findings and previous studies on pesticide poisoning conducted in Kuwait [18,19,20] all pointed to unsafe pesticide handling practices among farmers. Despite its limitation, this study provides an overview of pesticide safety knowledge and practices among farmers in Kuwait, and can contribute to educational and policy recommendations that aim at preventing or reducing the hazards associated with pesticides. The results may be relevant in other countries where greater and widespread use of pesticides among smallholder farmers is a dominant trend.

## 5. Conclusions

The adverse effects of pesticides have been widely documented. However, awareness among farmers and other applicators of the importance of protecting themselves and the environment from hazards associated with handling pesticides is still lacking. This study investigated the levels of knowledge and practices of Kuwaiti smallholder vegetable farmers regarding the safe use of pesticides. Our study reveal farmers lack adequate knowledge of pesticides, and often adopt risky behaviors when handling pesticides. Knowledge deficits included the poor use of PPE and other safety measures, and the improper storage and disposal of pesticides. To increase farmers’ knowledge about pesticide and limit the hazards associated with pesticides, it is recommended that priority is given to developing and implementing pesticide safety educational and certification programs for farmers. Where provided, the training must address health effects associated with exposure to pesticides, the effects of pesticides on the environment, improvements in disposal and storage of pesticides, pesticide risk reduction strategies, and understanding of the pesticide regulatory framework in Kuwait. Training pesticide retailers to increase their knowledge of pesticides is also critical, since they are farmers’ primary source of information about pesticides. Finally, intervention strategies by regulatory agencies to strengthen enforcement mechanisms of current pesticide laws, through regular surveillance and monitoring pesticide safety compliance at the retail and farm level is a necessity in promoting safe pesticide use. In addition, the importation, sale and the use of highly hazardous pesticides should be restricted.

## Figures and Tables

**Table 1:** Classification of pesticides used by farmers and their toxicological class.

Active Ingredient	WHO Toxicity Class ^a^	Classification by Main Group
Primiphos methyl	II	Organophosphate
Diazinon	II	Organophosphate
Acephate	II	Organophosphate
Monocrotophos	Ib	Organophosphate
Malathion	III	Organophosphate
Profenofos	II	Organophosphate
Trichlorfon	II	Organophosphate
Dimethoate	II	Organophosphate
Dimethoate	II	Organophosphate
Fenvalerate	II	Pyrethroid
Lambda-cyhalothrin	II	Pyrethroid
Permethrin	II	Pyrethroid
Cypermethrin	II	Pyrethroid
Beta-cyfluthrin	Ib	Pyrethroid
Etofenprox	III	Pyrethroid
Fenpropathrin	II	Pyrethroid
Deltamethrin	II	Pyrethroid
Bifenthrin	II	Pyrethroid
Alpha-cypermethrin	II	Pyrethroid
Benfuracarb	II	Carbamate
Methomyl	Ib	Carbamate
Oxamyl	Ib	Carbamate
Acetamiprid	II	Neonicotinoid
Thiacloprid	II	Neonicotinoid
Imidacloprid	II	Neonicotinoid
Spirotetramat derivative	III	Tetramic acid
Dinobuton	II	Dinitrophenol derivative
Spinosad	III	Spinosyns
Abamectin	Ib	Avermectin
Fenpyroximate	II	Fenpyroximate
Cyromazine	III	Cyromazine
Bifenazate	U	Bifenazate
Thiocyclam hydrogen oxalate	II	Nereistoxin
Bromopropylate	NC	Bromopropylate
Amitraz	II	Amitraz
Cartap hydrochloride	II	Nereistoxin
Flufenoxuron	III	Benzoylurea
Fipronil	II	Phenylpyrazole
Buprofezin	III	Buprofezin
Chlorfenapyr	II	Chlorfenapyr
Pyriproxyfen	U	Pyriproxyfen
Azoxystrobine	U	Fungicide and bactericides
Difenoconazole	II	Fungicide and bactericides
Mefenoxam	NC	Fungicide and bactericides
Triphenyltin hydroxide	U	Fungicide and bactericides
Propamocarb hydroxide	U	Fungicide and bactericides
Benomyl	U	Fungicide and bactericides
Dazomet	II	Fungicide and bactericides
Tebuconazole	II	Fungicide and bactericides
Hymexazol	III	Fungicide and bactericides
Tolclofos-methyl	U	Fungicide and bactericides
Maneb	Us	Fungicide and bactericide
Cuprous oxide	II	Fungicide and bactericides
Fenhexamid	U	Fungicide and bactericides
Mancozeb	U	Fungicide and bactericides
Copper hydroxide	II	Fungicide and bactericides
Copper oxychloride	II	Fungicide and bactericides
Thiophanate-methyl	U	Fungicide and bactericides
Dinocap	II	Fungicide and bactericides
Propamocarb	U	Fungicide and bactericides
Metalaxyl	II	Fungicide and bactericides
Fenamiphos	Ib	Nematicides
Metam-sodium	II	Nematicides
Oxamyl	Ib	Nematicides
Benomyl	U	Nematicides
Triazophos	Ib	Nematicides
Carbosulfan	II	Nematicides
Diamidafos	NC	Nematicides
Fenamiphos	Ib	Nematicides
Thionazin	NC	Nematicides
Fensulfothion	NC	Nematicides
Dichlofenthion	NC	Nematicides
Pendimethalin	II	Herbicides
Glyphosate	III	Herbicides
Oxadiazon	U	Herbicides
Metolachor	III	Herbicides
Sethoxydim	III	Herbicides

^a^ Ib: Highly hazardous; II: Moderately hazardous; III: Slightly hazardous; U: Unlikely to pose an acute hazard in normal use; NC: Not classified.

**Table 2:** Farmers’ knowledge, attitude and understanding about pesticide (n = 250).

Question	Variable	n	%
Do you think that pesticides affect human health?			
	Strongly agree	120	48
	Agree	57	23
	Disagree	60	24
	Strongly disagree	13	5
Do you think that pesticides affect the environment?			
	Strongly agree	98	39
	Agree	65	26
	Disagree	60	24
	Strongly disagree	27	11
Do you think pesticides are indispensable for high crop yield?			
	Strongly agree	155	62
	Agree	45	18
	Disagree	36	14
	Strongly disagree	14	6
Do you read, understand and follow pesticide labels?			
	Yes	70	28
	No	180	7
How do pesticides enter the human body? ^a^			
	Dermal	135	54
	Inhalation	215	86
	Oral	105	42
	Eye contact	65	26
	Don’t know	35	14
Do you know some pesticides are banned or restricted for use?			
	Yes	145	58
	No	105	42
Do you know the pesticides that are banned or restricted for use?			
	Yes	29	20
	No	116	80
Do you know the reasons for banning or restricting pesticides? ^a,b^			
	Highly toxic	84	58
	Not effective	115	79
	Expensive	41	28
	Dont’t know	30	21

^a^ Multiple responses allowed; ^b^ Percentage of respondents who knew that pesticides are banned or restricted for use (n = 145).

**Table 3:** Farmers’ practices on storage and disposal of pesticides (n = 250).

Question	Variable	n ^a^	%
Where do you store pesticides?			
	Open shed just for pesticides	86	34
	Refrigerator, with other items	20	8
	In the open field	76	30
	Locked chemical store	148	59
	Living area	50	20
	Animal house	38	15
What do you do with the unused leftover (mixed, diluted) pesticides?			
	Dispose in the field	87	35
	Mix only needed pesticides	45	18
	Apply on other crops	205	82
	Dispose in sewer	10	4
	Hazardous waste collection sites	57	23
What do you do with old pesticides stocks?			
	Return to retailer	12	5
	Hazardous waste collection sites	40	16
	Dispose in the field	68	27
	Buy only amount needed	155	62
	Dispose in sewer	7	3
What do you do with empty pesticide containers?			
	Discard on-farm	67	27
	Place in trash or dumpster	125	50
	Incinerate on-farm	108	43
	Hazardous waste collection sites	98	39
	Bury on-farm	63	25
	Reuse for other purposes	15	6

^a^ Multiple responses allowed.

**Table 4:** Farmers’ reported use of personal protected equipment when mixing and spraying pesticides to prevent occupational pesticide exposure (n = 150) ^a^.

Protective Equipment	Variable	n	(%)
Coveralls	Always	19	18
	Sometimes	15	14
	Never	71	68
Protective boots	Always	20	19
	Sometimes	28	27
	Never	57	54
Glasses/goggles	Always	50	48
	Sometimes	23	22
	Never	32	30
Gloves	Always	64	61
	Sometimes	38	36
	Never	3	3
Respirator	Always	4	4
	Sometimes	27	26
	Never	74	70
Hat	Always	44	42
	Sometimes	54	51
	Never	7	7

^a^ Number of respondents who wore at least one personal protective equipment during mixing and spraying pesticides.

**Table 5:** Farmers’ reported safety practices other than wearing personal protected equipment to prevent occupational pesticide exposure (n = 250).

Protective Equipment	Variable	n	%
Eating while mixing or spraying	Always	0	0
	Sometimes	70	28
	Never	180	72
Drinking while mixing or spraying	Always	0	0
	Sometimes	88	35
	Never	162	65
Smoking while mixing or spraying	Always	4	2
	Sometimes	98	39
	Never	148	59
Sprayed with the wind direction	Always	45	18
	Sometimes	90	36
	Never	115	46
Showering immediately after mixing or spraying	Always	205	82
	Sometimes	12	5
	Never	33	13
Wash work clothes separately	Always	45	18
	Sometimes	21	8
	Never	184	74

**Table 6:** Problems reported by farmers after mixing or spraying pesticides (n = 250) ^a^.

Symptoms	n ^b^	%
Headaches	205	82
Dizziness	102	41
Skin irritation	145	58
Nausea	122	49
Itchy eyes	198	79
Vomiting	25	10
Coughing	54	22
Shortness of breath	19	8
Excessive sweating	25	10
Fatigue	125	50
Stomach ache	10	4
Poor vision	6	3
No health impairment	45	18

^a^ Respondents were asked if within the past year prior to the date of the interview they experienced at least one heath impairment immediately after applying or handling pesticides; ^b^ Multiple responses allowed.
